# Opposite Effect of Cyclic Loading on the Material Properties of Medial Collateral Ligament at Different Temperatures: An Animal Study

**DOI:** 10.3389/fbioe.2022.925033

**Published:** 2022-06-14

**Authors:** Wentao Chen, Qing Zhou

**Affiliations:** State Key Laboratory of Automotive Safety and Energy, School of Vehicle and Mobility, Tsinghua University, Beijing, China

**Keywords:** pedestrian injury, knee joint, medial collateral ligament, temperature, cyclic loading, tensile properties

## Abstract

In traffic accidents, the medial collateral ligament (MCL) injury of the knee joint of pedestrians is common. Biofidelic material is important to realize MCL’s native biomechanics in simulations to clarify the injury mechanisms of pedestrians. Pedestrians’ MCLs usually experience cyclic loading at the intra-articular temperature of the knee joint before accidents. Temperature influences the material behaviors of ligaments. However, the mechanical properties of ligaments under cyclic loading have been widely evaluated only at room temperature rather than physiological temperature. Therefore, this study aimed to determine whether the difference between room and intra-articular temperatures influences the effect of cyclic loading on the mechanical properties of MCL. We measured the tensile properties of 34 porcine MCLs at room temperature (21–23°C) and intra-articular temperature (35–37°C), with either 10 cycles or 240 cycles of cyclic loading, a total of four different conditions. The structural responses and geometric data were recorded. After 240 cycles of cyclic loading, stiffness increased by 29.0% (*p* < 0.01) at room temperature and decreased by 11.5% (*p* = 0.106) at intra-articular temperature. Material properties were further compared because the geometric differences between samples were inevitable. At room temperature, after 240 cycles of cyclic loading, elastic modulus increased by 29.6% (*p* < 0.001), and failure strain decreased by 20.4% (*p* < 0.05). By contrast, at intra-articular temperature, after 240 cycles of cyclic loading, modulus decreased by 27.4% (*p* < 0.001), and failure strain increased by 17.5% (*p* = 0.193), insignificant though. In addition, there were no significant differences between the four groups in other structural or material properties. The results showed that temperature reversed the effect of cyclic loading on the mechanical properties of MCL, which may be caused by the high strength and thermally stable crosslinks of MCL. Therefore, for improving the fidelity of knee joint simulations and elucidating the injury mechanism of pedestrians, it is better to measure the mechanical properties of MCL at intra-articular temperature rather than room temperature.

## Introduction

The medial collateral ligament (MCL) injury of the knee joint of pedestrians is common in traffic accidents (Klinich, 2003; Steinmetz et al., 2020). MCL injuries cause economic burdens and seriously affect patients’ quality of life (Chakravarthy et al., 2007). To effectively protect pedestrians, it is important to elucidate the injury mechanism of MCL through experiments or simulations. Realizing the native biomechanical responses of MCL in simulations requires biofidelic material data (Cooper et al., 2019). However, because MCL materials are sensitive to the environment and loading history, it is essential to measure them under conditions close to the real working conditions.

As the name “pedestrian” suggests, pedestrians’ MCLs usually experience cyclic loading from a short-term walking process before traffic accidents. The influence of cyclic loading on the mechanical properties of ligaments or tendons (worth considering given the similarity to ligaments) has been widely studied. Su et al. (2008) tested the effect of 150 sinusoidal cyclic loading on the tensile properties of MCL and patellar tendon in rats. Both subjects showed a significant increase in failure stress, failure strain, and elastic modulus. Furthermore, Sekiguchi et al. (1998) and Teramoto and Luo (2008) compared the mechanical properties of rabbit anterior cruciate ligaments under different cyclic loading amplitudes and the mechanical properties of rat Achilles tendons under different cyclic loading durations, respectively. Their results indicated that appropriate cyclic loading conditions could improve mechanical strength. However, if the duration was too long or the amplitude was too large, cyclic loading may lead to fatigue damage and reduction of mechanical strength. Similar results were also found in cyclic fatigue loading of ligaments or tendons. Firminger and Edwards (2021) and Fung et al. (2010) studied the effect of cyclic fatigue loading using human and rat patellar tendons, respectively. They found that modulus increased significantly in the early response and then decreased. However, Zitnay et al. (2020) and Wang et al. (1995) observed that the modulus of rat tail tendons decreased continuously under cyclic fatigue loading. The effect of cyclic loading on the mechanical properties of ligaments or tendons is still controversial.

Walking not only applies cyclic loading to MCL but also increases body temperature by accelerating metabolism. The normal intra-articular temperature of human knee joints approximates 32–33°C (Harris and McCroskery, 1974; Becher et al., 2008). However, most studies tested the effect of cyclic loading at room temperature (did not mention the specific temperature values) rather than at intra-articular temperature. This may be because some early literature found that temperatures below body temperature had little effect on the mechanical properties of ligaments or tendons (Rigby et al., 1959; Dorlot et al., 1980; Quapp, 1997). Nevertheless, those early studies mainly focused on the low strain region and paid less attention to injury-related failure parameters. Other studies have found a significant influence of temperature on the mechanical properties of ligaments and tendons (Walker et al., 1976; Woo et al., 1983; KarisAllen and Veres, 2020), although these studies did not consider cyclic loading.

Few papers have studied the effects of temperature and cyclic loading simultaneously. Haut and Powlison (1990) studied the influences of cyclic loading (20 cycles and 0 cycles) and temperature (room temperature and 37°C) on human patella tendons. Tangent modulus and failure stress at 37°C were higher than those at room temperature, while failure strain at 37°C was lower than that at room temperature. Due to the difficulty of obtaining the experimental subject, they only had three valid samples in each group. Meghezi et al. (2012) studied the influences of cyclic loading (10 cycles and 0 cycles) and temperature (23°C and 37°C) on the type I collagen gels extracted from rat tail tendons. They found that the modulus at 37°C was smaller than that at 23°C. However, collagen gels may not be suitable to represent natural tendons or ligaments. Moreover, both studies regarded cyclic loading as preconditioning rather than simulating physiological activities, so only a few cycles were applied.

It is still unclear whether temperature would change MCLs’ responses to cyclic loading. If so, when studying MCL injuries caused by vehicle collisions, it should be better to use materials subjected to cyclic loading at intra-articular temperature. This study aimed to answer this question by studying the influence of cyclic loading on the mechanical properties of MCL at different temperatures. We measured the mechanical properties of porcine MCLs under four different conditions. Structural properties (i.e., stiffness, failure force, and failure displacement) and material properties (i.e., modulus, failure stress, and failure strain) were examined and compared. To the best of our knowledge, this is the first study to simultaneously compare the effects of temperature and cyclic loading on the mechanical properties of MCL.

## Materials and Methods

### Specimen Preparation

The stifle joints of the hind legs of mature Bama miniature pigs (10–11 months) were purchased from Wujiang Tianyu Biotechnology Co., Ltd (Jiangsu, China) and stored at -35°C for no more than 1 month. Pigs were slaughtered according to the food production process of the slaughterhouse; therefore, the review of the research ethics committee was not required. Stifle joints were thawed overnight in normal saline water (i.e., 0.9% sodium chloride solution) at room temperature and dissected the following day to obtain the femur-MCL-tibia complex. The storage and unfrozen process do not affect the mechanical properties of ligaments (Moon et al., 2003; Moon et al., 2006; Jung et al., 2011). After that, we measured the geometric dimensions of the MCL. The cross-section was simplified as a rectangular shape (Woo et al., 1983), and its width and thickness were measured with a caliper at the midsubstance. The dimensions were averaged on three individual measurements. Dissection and measurement were performed at room temperature, and the ligament surface was kept moist by spraying normal saline water intermittently.

### Test Apparatus

**FIGURE 1:**
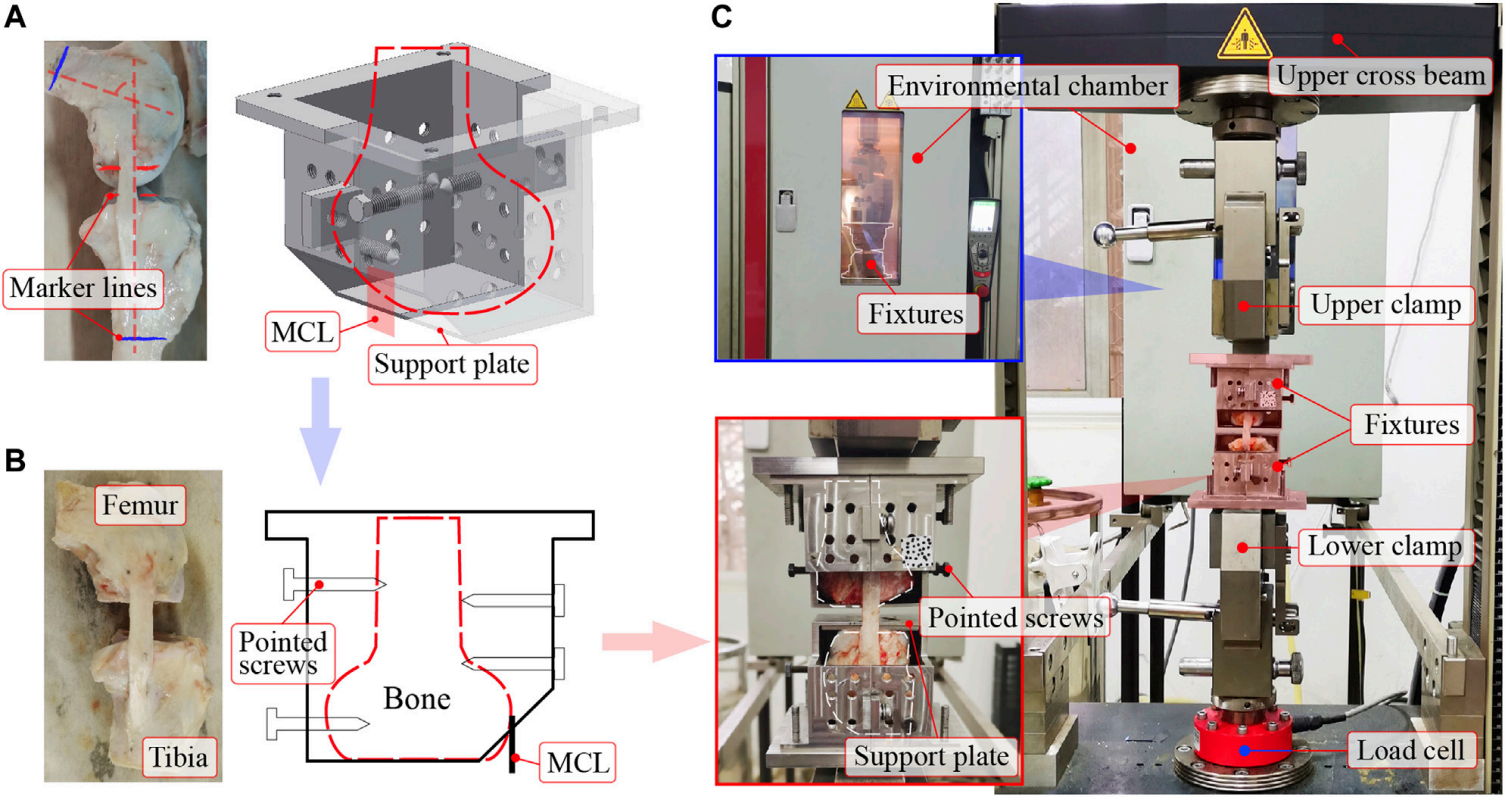
The femur-MCL-tibia complex mounted on a tensile apparatus. **(A)** Intact femur-MCL-tibia complex, and its condyle placed in a custom-made fixture (before sawing). The red parallel marker lines indicate the contact surfaces between the condyles and support plates. The blue marker lines mark the maximum allowable bone length that the fixtures can entirely wrap. **(B)** Osteotomized femur-MCL-tibia complex, and its condyle fixed in the fixture by pointed screws (after sawing). The pointed screws were screwed into the condyle through thread holes on the sidewalls of the fixture. **(C)** The fixtures were clamped by the upper and lower clamps of the testing machine after fixing the femur-MCL-tibia complex in the fixtures. An environmental chamber was used to control the experimental temperature. MCL = medial collateral ligament.

Custom-made fixtures clamped the femur-MCL-tibia complex on a testing machine (Figure 1). The condyle was housed by the two symmetrical parts of the fixture (Figure 1A). The ligament substance passed through the hole near the support plate. To prevent the condyle from rotating around the articular surface, the condyle was sawed flat using a table saw and placed on the support plate, forming a plane-to-plane contact (Figure 1B). Moreover, the plane-to-plane contacts between the condyles and support plates can maintain a constant knee flexion angle during loading. The experimental knee flexion angle was 80° to ensure the even tautness of the fibers (Germscheid et al., 2011). At this flexion angle, red marker lines, which indicated the contact surfaces between the condyles and support plates, were drawn as parallel as possible (Figure 1A). Blue marker lines were drawn to mark the maximum allowable bone length that the fixtures can entirely wrap (Figure 1A). Then, the condyles were carefully sectioned along the marker lines to form the plane-to-plane contacts with the support plates. Subsequently, the position and orientation of the condyles on the support plates were carefully adjusted to a desirable lineup (i.e., ligament substance was vertical in both front and side views). The condyles were fixed at the desirable lineup by screwing pointed screws into them through the threaded holes on the sidewalls.

Tensile tests were executed on a Zwick Z020 TE testing machine (Figure 1C). A commercial load cell (precision: ± 0.5%) at the lower clamp measured the tensile force, and the motion of the upper cross beam recorded the displacement. An environmental chamber was used to control the ambient temperature by continuously blowing hot air. The ligament surface was kept moist throughout the experiment by intermittently spraying normal saline (i.e., excess saline was dripping from the ligament).

### Test Protocol

**FIGURE 2:**
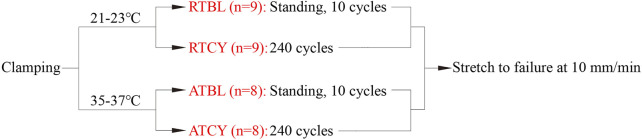
Test protocols of the four groups. Once the ambient temperature reached their respective specified values, 240 cycles of cyclic loading were applied to the samples of the RTCY and ATCY groups. The samples of the RTBL and ATBL groups were stood in the same temperature environment for the same time as the samples of the RTCY and ATCY groups, respectively, to keep the same temperature balance time; then, 10 cycles of cyclic loading were applied. After cyclic loading, all samples were stretched to failure at 10 mm/min.

To study the effect of temperature on MCL’s responses to cyclic loading, we carried out four groups of tests with different experimental conditions (Figure 2): room temperature environment and 10 cycles of cyclic loading (Room Temperature Baseline, RTBL), room temperature environment and 240 cycles of cyclic loading (RTCY), intra-articular temperature environment and 10 cycles of cyclic loading (ATBL), intra-articular temperature environment and 240 cycles of cyclic loading (ATCY).

The room temperature was 21–23°C, which was the room temperature on the days when we did the tests without any temperature control device. We assumed that the temperature difference between the rectum and knee joint of pigs is the same as that of humans (about 3°C). The porcine intra-articular temperature was then estimated to be 35–37°C, considering that porcine rectal temperature is about 38–39.5°C. Hence, the intra-articular temperature was set to 35–37°C. The cyclic loading parameters of the four groups were the same except for the number of cycles. The loading amplitude and frequency were 4% strain and 1.9 Hz, respectively, consistent with people’s normal walking (Pachi and Ji, 2005; Liu et al., 2011; Saunier et al., 2011; Willinger et al., 2020). Ten cycles of cyclic loading were used as preconditioning to stabilize the mechanical responses of the ligaments. It is the most commonly used number of cycles for preconditioning in the literature (Ebrahimi et al., 2019). To simulate a short-term walking process, 240 cycles of cyclic loading (approximately 2 min) were selected, which may be common in transfer scenes and has practical significance. Moreover, excessive cycles could lead to fatigue damage and weaken ligament strength. The 240 cycles of cyclic loading can keep the samples from fatigue damage that may affect subsequent tension (Teramoto and Luo, 2008). After cyclic loading, the specimen was ramped into failure at 10 mm/min (strain rate between 0.003 and 0.005 s^−1^) from a preload of 2 N.

### Data Processing and Analysis

**FIGURE 3:**
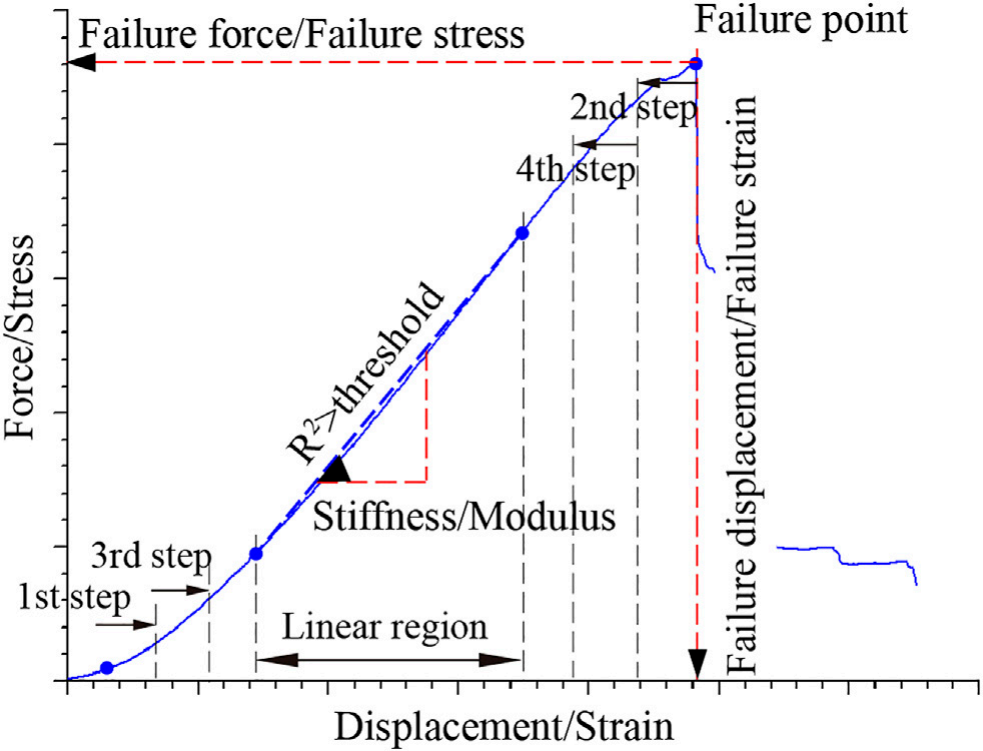
A typical tensile curve of MCL. The maximum force/stress point was defined as the failure point, and the corresponding abscissa and ordinate were the failure displacement/failure strain and failure force/failure stress, respectively. *R* ^2^ is the coefficient of determination of linear regression. Gradually reduce the number of data points for linear regression. Once *R* ^2^ was greater than a threshold (e.g., 0.99), the data segment used for this linear regression was defined as the linear region. The slope of the linear region was the stiffness/modulus.

The measured force-displacement curves were transformed into stress-strain curves by dividing the force by the initial cross-sectional area (i.e., engineering stress) and the displacement by the initial length (i.e., engineering strain), respectively. Failure point was the maximum force/stress point, and failure displacement/failure strain and failure force/failure stress were the abscissa and ordinate of the failure point, respectively (Figure 3). Stiffness/modulus was the slope of the linear region of the response curves. The linear region was calculated as follows. Data points from a starting point to an ending point were selected along the curve for initial linear fitting. The initial starting point (e.g., 2% strain) was a data point slightly beyond the toe region to eliminate the toe region’s influence on the regression. The initial ending point was the failure point. Thereafter, in a certain increment, we moved the starting and ending points toward the middle of the curve to perform new linear regression. This process gradually reduced the number of data points used for each new linear regression and was repeated until the coefficient of determination (i.e., *R* ^2^) reached 0.99. The data points used for the final linear regression were defined as the linear region.

All data were presented as mean ± standard deviation since nearly all data were normally distributed or only had a minor deviation from the normal distribution (Shapiro-Wilk tests). Intergroup stiffness and modulus were compared using two-way ANOVA. The response curve of MCL has a long linear region before reaching the failure point. The greater the failure displacement/failure strain, the higher the failure force/failure stress. Namely, they are related to each other. So, two-way MANOVA was used to compare failure displacement and failure force, as well as failure strain and failure stress. Finally, failure mode was treated as a categorical variable and compared using chi-square test. Results were statistically significant when the significance level (i.e., *p*-value) was smaller than 0.05.

## Results

**FIGURE 4:**
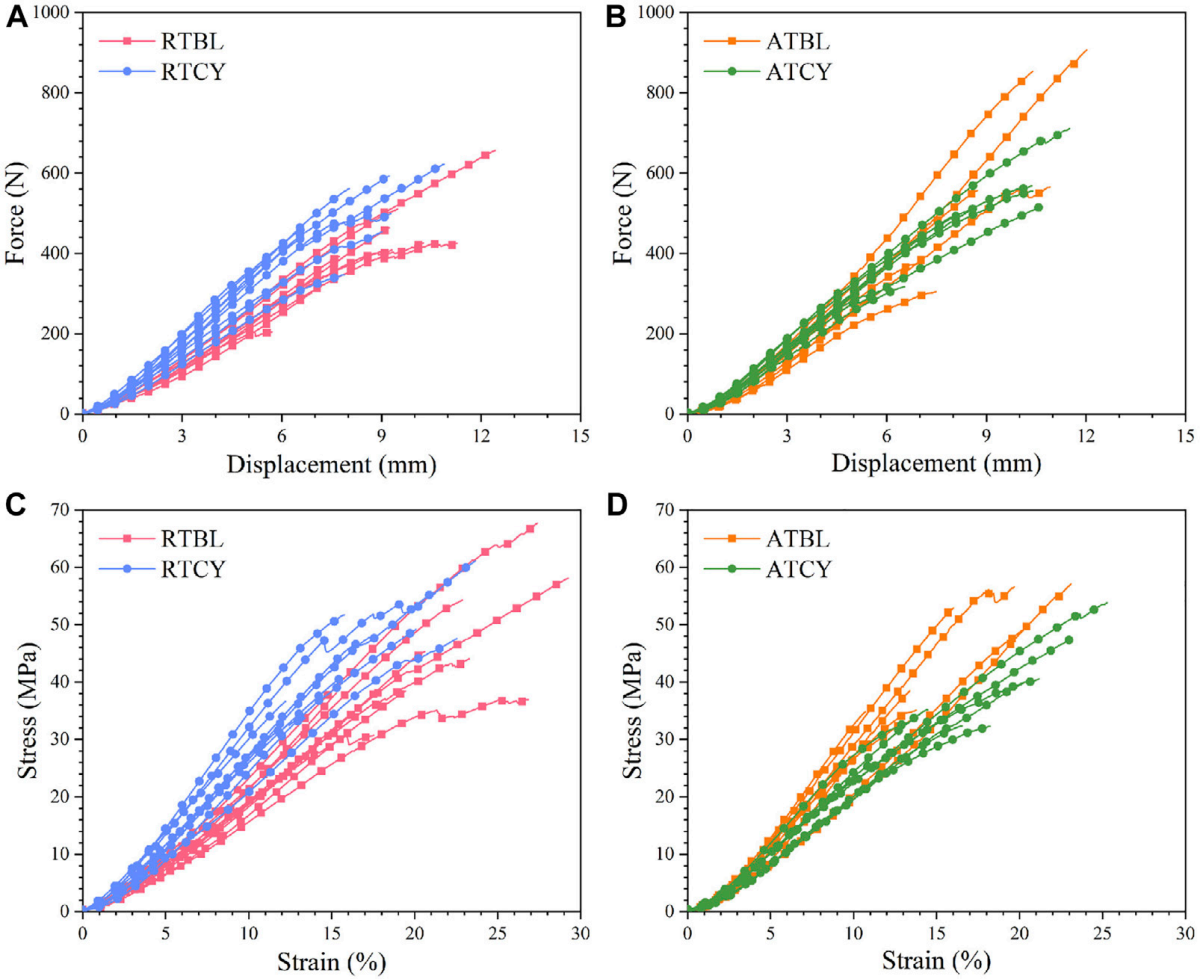
Tensile curves as a function of cyclic loading at two different temperatures. Force-displacement curves: **(A)** At room temperature (n = 9); **(B)** At intra-articular temperature (n = 8). Stress-strain curves: **(C)** At room temperature (n = 9); **(D)** At intra-articular temperature (n = 8). The curves end at their respective failure points. At room temperature, the average stiffness and modulus of the samples after 240 cycles of cyclic loading (i.e., RTCY) were higher than those of the samples only went through 10 cycles of cyclic loading (i.e., RTBL). At intra-articular temperature, the samples after 240 cycles of cyclic loading (i.e., ATCY) generally had lower stiffness and modulus than those after 10 cycles of cyclic loading (i.e., ATBL).

The RTBL and RTCY groups had nine samples each, and the ATBL and ATCY groups had eight samples each, a total of 34 valid data. Cyclic loading had different effects on the mechanical responses of the MCLs at room and intra-articular temperatures (Figure 4). Although the main effect of temperature or cyclic loading was insignificant, the interaction effect of temperature and cyclic loading significantly influenced the stiffness (*p* = 0.001). At room temperature, stiffness significantly increased by 29.0% (RTBL 52.26 ± 6.58 N/mm, RTCY 67.39 ± 10.43 N/mm; *p* < 0.01) after cyclic loading. While at intra-articular temperature, stiffness decreased by 11.5% (ATBL 69.27 ± 13.13 N/mm, ATCY 61.31 ± 6.63 N/mm; *p* = 0.106) after cyclic loading, insignificant though. There were no significant differences in failure displacement and failure force between the four groups.

We only observed substance rupture and tibial avulsion failure modes in the experiments, in line with Germscheid et al. (2011). There was no femoral avulsion, probably because the porcine samples were young. In young samples, the direct insertion of femoral attachments was stronger than the indirect insertion of tibial attachments (Thambyah et al., 2014). The failure mode ratio (i.e., substance rupture over tibial avulsion) was compared by chi-square test. There was no significant difference in failure mode ratio between groups (RTBL 4/5, RTCY 3/6, ATBL 2/6, ATCY 3/5; *p* = 0.960).

**FIGURE 5:**
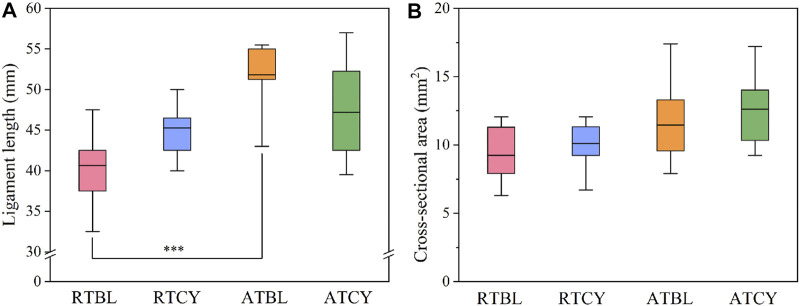
Geometric data of each group: **(A)** Ligament length; **(B)** Cross-sectional area. The RTBL and ATBL groups had a significant difference in ligament length (***p < 0.001). The box shows the mean ± interquartile range, and the whiskers show the minimum/maximum values.

**FIGURE 6:**
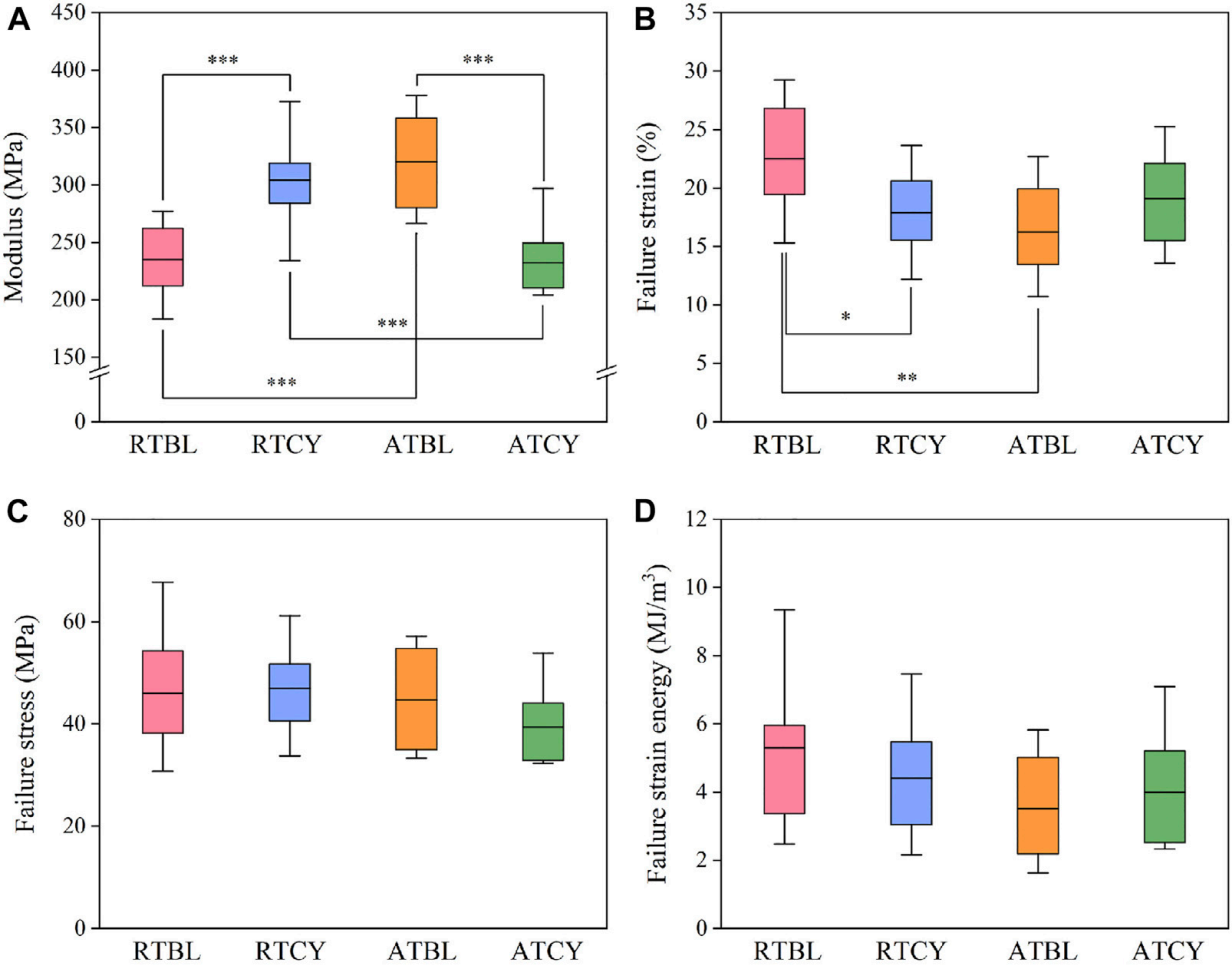
Material properties of each group: **(A)** Modulus; **(B)** Failure strain; **(C)** Failure stress; **(D)** Failure strain energy. The box shows the mean ± interquartile range, and the whiskers show the minimum/maximum values. Significant differences are indicated as: *p < 0.05, **p < 0.01, ***p < 0.001.

Differences in geometry are unavoidable, especially in ligament length (Figure 5). To eliminate the influence of ligament geometry, we then compared the material properties (Woo et al., 1993). Similar to the results of stiffness, the main effect of temperature or cyclic loading was insignificant; in contrast, the interaction effect significantly influenced the modulus (*p* < 0.001) (Figure 6A) and failure strain (*p* = 0.017) (Figure 6B). Modulus increased by 29.6% (RTBL 234.87 ± 32.18 MPa, RTCY 304.38 ± 39.59 MPa; *p* < 0.001) and failure strain decreased by 20.4% (RTBL 22.50 ± 4.72%, RTCY 17.90 ± 4.03%; *p* = 0.030) after 240 cycles of cyclic loading at room temperature. Meanwhile, modulus decreased by 27.4% (ATBL 320.23 ± 43.31 MPa, ATCY 232.42 ± 33.89 MPa; *p* < 0.001) and failure strain increased by 17.5% (ATBL 16.24 ± 4.17%, ATCY 19.09 ± 4.13%; *p* = 0.193) after 240 cycles of cyclic loading at intra-articular temperature. No significant difference was found in failure stress (*p* = 0.362) (Figure 6C). Since the failure strain and modulus changed inversely, we used two-way ANOVA to compare the failure strain energy (i.e., the area under the stress-strain curve from the origin to the failure point). The result showed no significant difference in failure strain energy between groups (Figure 6D).

## Discussion

MCL mainly functions at intra-articular temperature (close to body temperature) rather than room temperature, especially when considering warm-up activities. We observed an opposite effect of cyclic loading on the mdulus and failure strain of MCL at room temperature and intra-articular temperature. Therefore, we advise using the mechanical properties of MCL at intra-articular temperature to simulate pedestrian injury. Moreover, we compared our results with the literature to figure out the possible mechanisms of the opposite effect.

### Mechanism of Temperature Effect

We first analyzed the individual effect of temperature based on the results of the RTBL and ATBL groups. Modulus increased, and failure strain decreased significantly with the increase of temperature (Figure 6). Our results were similar to those of Haut and Powlison (1990), using human patella tendons as subject, but contrary to the results of Rigby et al. (1959), Walker et al. (1976), Meghezi et al. (2012), and KarisAllen and Veres (2020). Rigby et al. (1959) tested rat tail tendons at different temperatures and found that increased temperature hastened tendon breakdown when the tensile strain exceeded about 4%. Meghezi et al. (2012) tested the type I collagen gels extracted from rat tail tendons and found a significant decrease in modulus with the temperature increase from 23°C to 37°C. KarisAllen and Veres (2020) tested the tensile properties of bovine tail tendons at 24°C, 37°C, and 55°C. A decreasing trend in modulus and an increasing trend in failure strain can be seen when temperature increase from 24°C to 37°C. The comparison inspired us to focus on the differences in experimental subjects.

**FIGURE 7:**
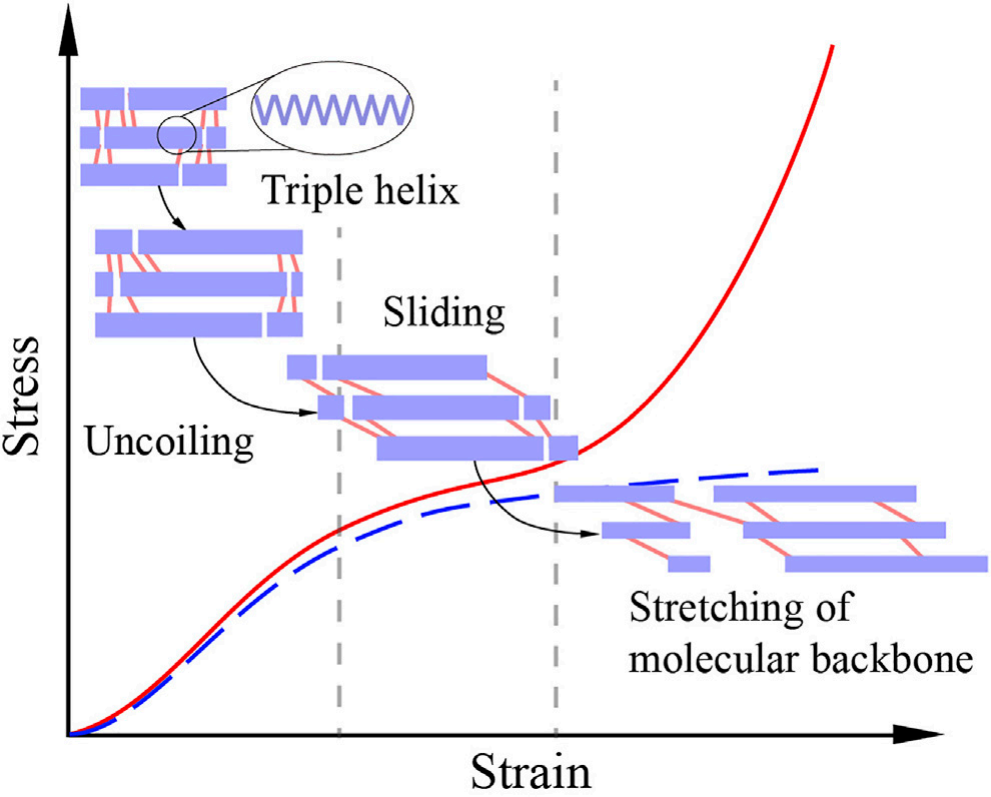
The stress-strain response of a crosslinked collagen fibril under unidirectional stretching. The purple bars represent collagen molecules, and the pink lines represent crosslinks. Collagen molecules first uncoil their triple helix structures in the uncoiling stage. Then, a relatively gentle sliding stage is formed due to the sliding of molecules. Crosslinks also start to work in the sliding stage, restraining sliding and enhancing mechanical properties. If the strength of the crosslinks is weaker than that of the molecular backbones, the crosslinks will fail in the sliding stage, resulting in a two-stage response (the blue dot line). Otherwise, the molecular backbones will be stretched and generate a sharply rising curve segment, resulting in a three-stage response (the red line) (Svensson et al., 2013; Depalle et al., 2015).

Patellar tendon and tail tendon have different mechanical responses at the fibril level (Svensson et al., 2013; Depalle et al., 2015). Under uniaxial tension, a fibril firstly uncoils the triple helix structures of collagen molecules, resulting in a gradual increase in stress. As the stress increases, collagen molecules begin to slide relatively, forming a slowly increasing stress. The sliding between collagen molecules is restrained by intermolecular crosslinks (chemical bonds). The crosslinks of the fibril of patellar tendon have sufficiently high strength, so the molecular backbones are stretched at the end of the sliding stage, giving a sharp stress rising. Thus, the fibril of patellar tendon experiences a three-stage response under unidirectional stretching (Figure 7). On the contrary, in tail tendon fibrils, the strength of crosslinks is weaker than that of molecular backbones. Therefore, the molecular backbones will not be stretched, and the mechanical response of tail tendon fibrils has only the first two stages (Figure 7). In addition to the difference in crosslink strength, patellar tendon and tail tendon are also different in crosslink type. The crosslinks of patellar tendon are more thermally stable than those of tail tendon (Svensson et al., 2013; Herod et al., 2016; KarisAllen and Veres, 2020). Meanwhile, patellar tendon and knee ligaments (including MCL) have similar collagen crosslink types and concentrations (Hanada et al., 2014). Therefore, we believe that the fibrils of MCL and patellar tendon have similar three-stage responses. Moreover, the crosslinks of MCL are also more thermally stable than those of tail tendon.

Due to the thermal stability, the temperature of 37°C is not sufficient to break the crosslinks of MCL fibrils. The strength of the sliding and molecular stretching stages may be almost unchanged when temperature rises from room temperature to intra-articular temperature. However, the rising temperature can partially break the hydrogen bonds and melt the triple helix structures of collagen molecules, which leads to softening in the uncoiling stage (Leikina et al., 2002; Trębacz and Wójtowicz, 2005). Hence, the 2 N preload applied to MCL tissue in the experiments eliminated more fibril level coiling at intra-articular temperature than at room temperature. Consequently, molecular backbones were stretched at a lower strain value at intra-articular temperature than at room temperature. The “accelerated” stretching of the molecule backbones at intra-articular temperature may contribute to the higher modulus and lower failure strain in the ATBL group than in the RTBL group. Compared with MCL, the heat-labile crosslinks of tail tendon experience extensive failure with the increase of temperature, leading to an increase in the sliding of collagen molecules and a reduction of the fibril bearing capacity. As a result, modulus at the tissue level decreases after temperature rise. Moreover, fewer crosslinks allow greater sliding, increasing the failure strain.

This analysis can explain the differences between this study and most of the literature, except Walker et al. (1976). Walker et al. (1976) tested canine patellar tendons and Achilles tendons at different temperatures. Although not significant, they found that the modulus of patellar tendon decreased with temperature increase, opposite to the analysis above. The conflict may be caused by their temperature control method. They first immersed the subjects in 50°C water and then mounted them on the testing machine. Load-deflection curves at different temperatures were obtained during natural cooling. The 50°C is much higher than normal body temperature, at which even thermally stable crosslinks may fail. Once the strength of the remaining crosslinks is weaker than that of molecular backbones, the fibril response of patellar tendon will become a two-stage curve similar to that of tail tendon, and patellar tendon tissue will be weakened. In addition, it is not easy to balance the tendon temperature during natural cooling, which affects the reliability of their results (Walker et al., 1976).

### Crosslink and Cyclic Loading

The analysis above can also partially explain the controversy on the effect of cyclic loading on ligament or tendon properties in the literature, mainly tested at room temperature. Studies using tail tendons found a continuously weakened modulus during cyclic fatigue loading (Wang et al., 1995; Zitnay et al., 2020); while studies using MCLs (Su et al., 2008), patellar tendons (Su et al., 2008; Fung et al., 2010; Firminger and Edwards, 2021), and anterior cruciate ligaments (Sekiguchi et al., 1998) found strengthened mechanical properties after short-term cyclic loading (not involving fatigue), although the significantly strengthened physical quantities (e.g., modulus, failure stress) were different.

Cyclic loading can partially uncoil the triple helix structures of collagen molecules and destroy the crosslinks (Firminger and Edwards, 2021; Al Makhzoomi et al., 2022). Therefore, cyclic loading can weaken the fibril bearing capacity of tail tendon with low crosslink density. Comparatively, because molecular backbones govern the bearing capacity of MCL fibrils with high crosslink strength, a small amount of crosslink failure caused by cyclic loading will neither change the mechanical response of MCL fibrils nor reduce its bearing capacity. Moreover, similar to the temperature increase effect, micro uncoiling caused by cyclic loading and macro preload jointly accelerate the stretching of collagen backbones. Hence, appropriate cyclic loading duration can increase the modulus and reduce the failure strain of MCL, which is consistent with the results of the RTBL and RTCY groups.

### Interaction Effect of Temperature and Cyclic Loading

We finally analyzed why the effect of cyclic loading on MCL mechanical properties at intra-articular temperature was opposite to that at room temperature. Temperature increase or cyclic loading alone can partially destroy the intermolecular crosslinks and uncoil the triple helix structures of collagen molecules. When they are applied simultaneously, the number of failed crosslinks is more than that when temperature rise or cyclic loading is applied alone. If the number of failed crosslinks exceeds a certain threshold and the strength of the remaining crosslinks is weaker than that of molecular backbones, the molecular backbones will not be stretched. Consequently, the fibril bearing capacity of MCL will be reduced. This process may have occurred in the samples of the ATCY group, which experienced 240 cycles of cyclic loading at intra-articular temperature. Hence, the ATCY group had a lower modulus and a higher failure strain than the ATBL group.

Our explanation focused on the fibril level (i.e., crosslinks and collagen molecules) because fibrillar properties govern the mechanical response at the tissue level (Su et al., 2008; Svensson et al., 2012; Svensson et al., 2013; Firminger and Edwards, 2021). Although many studies have tried to find out the load transfer mechanism from fibril behavior to tissue response, no definite conclusions have been drawn (Redaelli et al., 2003; Robinson et al., 2005; Haraldsson et al., 2008; Fessel and Snedeker, 2009; Henninger et al., 2013; Al Makhzoomi et al., 2022). Therefore, at this stage, we cannot exclude any possible contribution of inter-fibril load transfer components to the mechanical response of ligaments or tendons, which may be found in the future. Inter-fibril changes and experimental errors may be potential reasons for the differences in statistically significant physical quantities. For example, Su et al. (2008) observed a significant increase in failure stress, failure strain, and modulus after cyclic loading at room temperature. Nevertheless, comparing the results of the RTBL and RTCY groups, we only found a significant increase in modulus after cyclic loading. Further experiments are needed to observe the crosslink changes under different conditions to verify the explanation.

### Limitations

This study has a few limitations. First, the experimental conditions mixed human and pig parameters. We applied human walking parameters to the cyclic loading of porcine samples due to the lack of porcine “walking” data. In addition, the porcine intra-articular temperature was estimated according to porcine rectal temperature and the temperature difference between human rectum and knee joint. The responses of human MCL to cyclic loading at intra-articular temperature need to be further measured. Second, MCL is stretched at a strain rate of 30–50 s^−1^ in 40 km/h pedestrian-automotive collisions (van Dommelen et al., 2005). However, this study only measured the mechanical responses of MCL under quasi-static state. Further high strain rate material tests of MCL at intra-articular temperature are necessary for pedestrian simulations. Third, the experimental matrix had only two levels each of temperature and cyclic loading, so the results only proved that temperature does influence the effect of cyclic loading on MCL, and it is reasonable to use the mechanical properties at intra-articular temperature for pedestrian simulations. Nevertheless, pedestrians’ walking time may range from minutes to hours. This study only measured the mechanical properties of MCL after 240 cycles of cyclic loading (approximately 2 min of walking). For simulating pedestrian injuries under impact loading, experiments at intra-articular temperature with other numbers of cycles may also be necessary. Forth, the hydration environment (in aqueous saline solution or air) also affects the mechanical properties of soft tissue (Meghezi et al., 2012; Grant et al., 2015). The environmental chamber raised the ambient temperature by blowing in hot air continuously, which may affect the moisture. Although we intermittently sprayed normal saline during the experiments to keep the ligament surface moist, whether the humidity maintenance method affects the results may require future study.

## Conclusion

The effect of cyclic loading on the tensile properties of MCL is influenced by temperature. Stiffness and modulus increased, and failure strain decreased after cyclic loading at room temperature. While at intra-articular temperature, cyclic loading influenced MCLs’ properties in the opposite way, decreasing the stiffness and modulus and increasing the failure strain. This may be caused by crosslink changes. Considering that material data is essential to improve the fidelity of knee joint simulations and understand the injury mechanisms of pedestrians, it is recommended to measure the mechanical properties of MCL at intra-articular temperature rather than at room temperature.

## Data Availability

The raw data supporting the conclusions of this article are available from the corresponding author on reasonable request.
